# Statins As Anti-Hypertensive Therapy: A Systematic Review and Meta-Analysis

**DOI:** 10.7759/cureus.57825

**Published:** 2024-04-08

**Authors:** Zahid Khan, Amresh Gul, Gideon Mlawa, Priyadarshini Bhattacharjee, Syed Aun Muhammad, Jonard Carpio, Hassan Yera, Maureen Wahinya, Axel P Kazeza, Mehul S Amin, Animesh Gupta

**Affiliations:** 1 Acute Medicine, Mid and South Essex NHS Foundation Trust, Southend-on-Sea, GBR; 2 Cardiology, Barts Heart Centre, London, GBR; 3 Cardiology and General Medicine, Barking, Havering and Redbridge University Hospitals NHS Trust, London, GBR; 4 Cardiology, Royal Free Hospital, London, GBR; 5 General Practice, Lifeline Hospital, Salalah, OMN; 6 Internal Medicine and Diabetes and Endocrinology, Barking, Havering and Redbridge University Hospitals NHS Trust, London, GBR; 7 Cardiovascular Medicine, University of South Wales, Cardiff, GBR; 8 General Internal Medicine, Cambridge University Hospitals NHS Foundation Trust, Cambridge, GBR; 9 School of Clinical Medicine, University of Cambridge, Cambridge, GBR; 10 Cardiology, Mid and South Essex NHS Foundation Trust, Southend-on-Sea, GBR; 11 Internal Medicine, Mid and South Essex NHS Foundation Trust, Southend-on-Sea, GBR; 12 Cardiology, The Shrewsbury and Telford Hospital NHS Trust, Shrewsbury, GBR; 13 Internal Medicine, Kenyatta University Teaching, Referral & Research Hospital, Nairobi, KEN; 14 Internal Medicine, Watu Wetu SAS, Kolwezi, COD; 15 Internal Medicine, Southend University Hospital, Southend-on-Sea, GBR; 16 Acute Internal Medicine, Mid and South Essex NHS Foundation Trust, Southend-on-Sea, GBR; 17 Acute Internal Medicine and Intensive care, Barking, Havering and Redbridge University Hospitals NHS Trust, London, GBR

**Keywords:** simvastati, rosuvastatin, systematic reviews of statins, randomized controlled trials, isolated systolic hypertension, systolic and diastolic blood pressure, antihypertensive therapy, hypertension and statins, hypertensive individuals on statins, systematic review and meta analysis

## Abstract

Hypertension is the most prevalent condition in clinical practice. Hypertension, diabetes, and hypercholesterolaemia are major contributing factors to cardiovascular diseases. They commonly coexist in a single patient. Statins have been used as prominent medicines for the reduction of cardiovascular events. Statins have been shown to reduce blood pressure in patients with hypertension and have lipid-lowering properties in recent articles. Statins reduce blood pressure because of their impact on endothelial function, their interactions with the renin-angiotensin system, and their influence on major artery compliance. This meta-analysis aimed to ascertain the effectiveness and efficacy of statins for managing hypertension in patients with hypertension. Systematic searches were conducted on PubMed, Science Direct, Embase, Cochrane Library, and Google Scholar. Randomized controlled trials, systematic trials, and cohort studies were retrieved using keywords on statins and their use in patients with hypertension. Exclusion criteria included studies that were not in the English language, studies that did not include patients on statins with hypertension, studies that did not provide enough information, technical reports, opinions, or editorials, and studies involving patients < 18 years old. The inclusion criteria were randomized controlled trials, meta-analyses, adult patients aged > 18 years old, and studies that were freely available or through institutional login. This meta-analysis scrutinized 9361 randomized controlled trials, clinical trials, meta-analyses, and systematic reviews, of which 32 articles including 25 randomized controlled trials and seven meta-analyses were included in the final analysis. This meta-analysis of the role of statins in hypertensive patients aimed to determine the outcome of hypertension control along with antihypertensive medication. Our study showed that statins are useful in reducing both systolic and diastolic blood pressure. We used a heterogeneous model for analysis due to variations in the study characteristics. The I2 value was 0.33 (0.76, 0.10) for systolic blood pressure and 0/88 (0.86, 0.90) for diastolic blood pressure. The I2 value for the seven meta-analyses included in the study was 1.79 (2.88, 0.69).

## Introduction and background

Essential hypertension, also known as high blood pressure (BP), is the most common disease in adults and the leading cause of death globally [1]. In the past few years, the prevalence of hypertension has surged significantly in low- and middle-income countries [1,2]. In 2008, approximately 40% of adults aged ≥ 25 years had hypertension [3]. Furthermore, hypertension is a major causative factor for at least 45% of deaths due to heart disease and 51% of deaths due to stroke worldwide [3-5]. Large cohort studies have provided evidence that hypertension is an imperative risk factor for heart valve disease, stroke, heart failure, myocardial infarction, kidney disease, atrial fibrillation, aortic disease, and dementia [1,6]. According to the guidelines of the European Society of Cardiology, hypertension is defined as systolic BP (SBP) ≥ 140 mmHg and/or diastolic BP (DBP)  ≥ 90 mmHg [7]. However, hypertension is further categorized as normal, high-normal, or grades 1-3 BP readings per office BP. Hypertension rarely occurs in isolation and is often accompanied by glucose intolerance and dyslipidaemia which are the major risk factors for cardiovascular diseases (CVDs) [7]. In addition to lifestyle modifications and non-pharmacological measures, including the DASH (dietary approach to stop hypertension) diet to control BP, the majority of patients also require pharmacological treatment. There are five major drug classes for lowering BP: angiotensin-converting enzyme inhibitors (ACEI), beta-blockers, calcium channel blockers, angiotensin receptor blockers, and diuretics [7,8]. However, there are some cases in which BP would be difficult to control despite all the medications or triple therapy [7,9].

Statins lower the amount of cholesterol synthesized in the liver by competitively inhibiting the enzyme 3-hydroxy-3-methylglutaryl coenzyme A (HMG-CoA) reductase, which is the rate-limiting step in cholesterol synthesis. Lower plasma concentrations of low-density lipoprotein (LDL) and other apolipoprotein B (ApoB)-containing lipoproteins, such as triglyceride (TG)-rich particles, are the results of the reduction in intracellular cholesterol, which also increases the expression of the LDL receptor (LDLR) on the surface of hepatocytes [10]. Statins reduce blood cholesterol levels and enhance endothelial function by preserving endothelial nitric oxide synthase, which causes vasodilation and prevents arterial disease development [11]. A previous study demonstrated that statins can prevent the progression of arterial stiffness and lower BP when combined with recommended antihypertensive treatment [11,12]. Multiple studies have supported a reduction in BP in hypertensive patients treated with statins and antihypertensive medication [13].

A recent meta-analysis studied the positive effects of combination statin use on cardiovascular risk in hypertensive patients [1,14,15], and our meta-analysis examined the impact of statin therapy (alone or in combination with other antihypertensive medications) on various outcomes associated with hypertension. Hypertension is one of the most important risk factors for endovascular atherosclerotic disease, and it increases the risk of cardiovascular atherosclerosis when combined with other risk factors. The combined use of statins and antihypertensive therapy has synergistic effects on the prevention of CVD progression. There is a lack of evidence supporting the use of statins with antihypertensive therapy in patients with grade 1 hypertension and normal cholesterol levels [16]. Several randomised controlled trials (RCTs) have demonstrated the positive effects of statins on BP; however, other studies have shown neutral or no effects [17-40]. This meta-analysis aimed to analyse previous RCTs and meta-analyses on the role of statins in patients with hypertension.

## Review

Materials and methods

Search Strategy

This study was registered with the International Prospective Register of Systematic Reviews (PROSPERO) and the National Institute for Health and Care Research (NIHR) under the registration number CRD42023493395. A meta-analysis was conducted using multiple search engines to determine the role of statins in patients with hypertension. The literature search was performed on Cochrane Library, PubMed, Science Direct, Embase, and Google Scholar. Medical Subject Headings (MeSH) terms used for the search were “statin”, "hypertension", “statins in hypertension”, “the role of statins in hypertensive patients ‘, “statins in high blood pressure’, “ Statins in various grade of hypertension‘, “hypertension and statins, and “statin and blood pressure lowering effect”, “ atorvastatin", “rosuvastatin”, “pravastatin”, and “simvastatin”, "statins as antihypertensive therapy", "combined statin and antihypertensive therapy". A combination of these MeSH terms was used in the literature search. A total of 9361 articles were identified. After removing duplicates and articles that did not meet the inclusion and exclusion criteria, 25 RCTs and seven meta-analyses were included in the final review. Two independent reviewers performed a literature search using the above search engines and agreed on the inclusion and exclusion of articles. In case of disagreement between the two reviewers, a third independent reviewer’s opinion was sought, and a decision about the inclusion or exclusion of the articles was made through a majority consensus [1-48].

The study was performed by following the PICO (patient/population, intervention, comparison and outcomes) model (Table 1).

**Table 1:** Inclusion and exclusion criteria for eligible studies based on the PICO model PICO: patient/population, intervention, comparison, and outcomes

Study characteristic	Inclusion criteria	Exclusion criteria
Population	Adult patients (aged 18 years and older) of any gender with hypertension and with or without hypercholesterolaemia	Patients <18 years of age, patients without hypertension and with or without hypercholesterolaemia
Intervention	Hypertensive patients who received statins therapy with or without antihypertensive medications	Hypertensive patients who received neither statins nor placebo therapy with or without antihypertensive medications
Comparator	Hypertensive patients who received placebo therapy with or without antihypertensive medications	Hypertensive patients who did not receive placebo therapy with or without antihypertensive medications
Outcomes of interest	Primary: Change in systolic and diastolic blood pressure. Secondary: Endothelial function, inflammatory response and lipid level response.	No primary or secondary outcomes of interest are reported
Study design	Randomized controlled trials or systematic reviews and meta-analyses	Cohort studies, case reports, case series, editorials, cross-sectional studies
Publications	English language studies	Studies published in languages other than English

Population: Studies such as RCTs, systematic reviews, and meta-analyses involving patients with hypertension, aged > 18 years, and any gender were included in this study.

Intervention: Studies such as RCTs, systematic reviews, and meta-analyses involving patients with hypertension, aged > 18, and who received statin therapy alone or in combination with anti-hypertensive medications were included in this study.

Comparison: Studies such as RCTs, systematic reviews, and meta-analyses involving patients with hypertension, aged > 18, and who received placebo therapy alone or in combination with anti-hypertensive medications were included in this study.

Outcome: The primary outcome of interest in this study was the effect of statin on SBP and DBP. Secondary outcomes included endothelial function, inflammatory response and lipid level response.

Timeline of the Study

A literature search was carried out to identify studies conducted from 21 December 2023 to 31 January 2024.

Criteria for Selection

We included studies that were systematic reviews, meta-analyses, or RCTs. Articles were included only if the intervention was either statin as a solo treatment or a combination therapy of statin and antihypertensive, focusing on the role of statins in lowering SBP and DBP in patients with hypertension. Articles were selected based on pre-specified inclusion and exclusion criteria. The inclusion criteria were studies written in English only, studies providing sufficient information to calculate the odds ratio, relative risk, or mean difference for participants, studies including adults aged > 18 years, and studies from the year 2000 onwards. We included only RCTs, systematic reviews, and meta-analyses in this study. Studies that were not readily available freely or through institutional logins, that included patients aged < 18 years, and were not written in English were excluded. Case reports, editorials, opinion articles, cohort studies, cross-sectional studies, and case series were excluded. We also excluded studies that focused on the use of statins in pulmonary hypertension and portal hypertension with liver cirrhosis. Statin studies, including RCTs and meta-analyses, that did not provide sufficient data about hypertension and missing data were also excluded from the final analysis.

Data Extraction

Data extraction was performed by two independent reviewers and included relevant demographic information, such as first author and year of publication, study design, study period, sample size, mean age or years, patient female/male, experimental intervention, and outcome, that were collected from eligible studies. The data were cross-checked by a third independent reviewer and any differences were resolved through discussion.

Risk of Bias Assessment

The Grading of Recommendations, Assessment, Development, and Evaluations (GRADE) classification system was used to assess the overall certainty of the body of evidence for each outcome across both the systematic reviews and RCTs [18]. The risk of bias in the included studies was assessed using the revised Cochrane Collaboration Risk of Bias (RoB 2) tool. The evaluations included (i) random sequence generation, (ii) allocation concealment, (iii) blinding of subjects and researchers, (iv) blinding of outcome measurements, (v) incomplete outcome data, (vi) selective outcome reporting, and (vii) other prejudices [16].

Quality of Evidence Assessment

**Figure 1:**
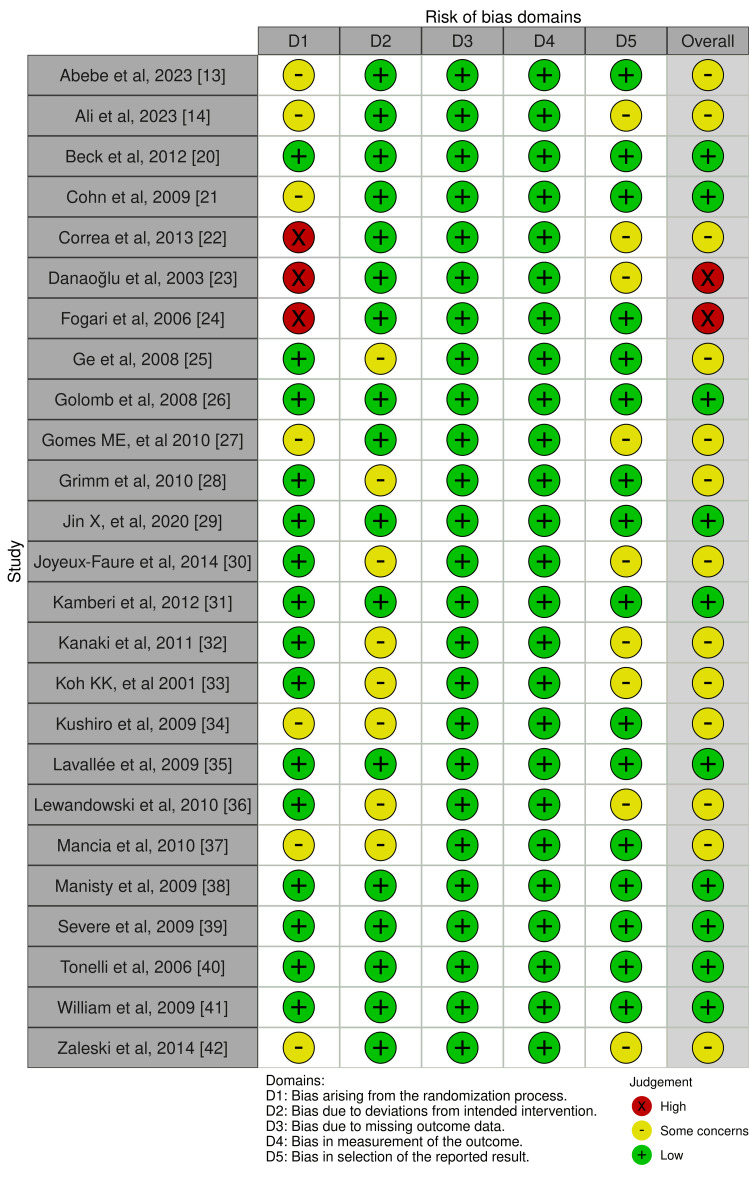
RoB 2 tool for the randomized controlled trials included in the systematic review RoB 2: Risk of Bias 2 References: [13,14,20-42]

**Figure 2:**
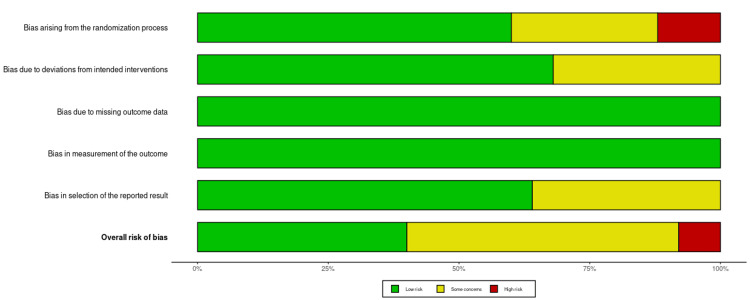
RoB 2 risk of bias assessment summary plot for the randomized controlled trials included in the meta-analysis RoB 2: Risk of Bias 2

**Figure 3:**
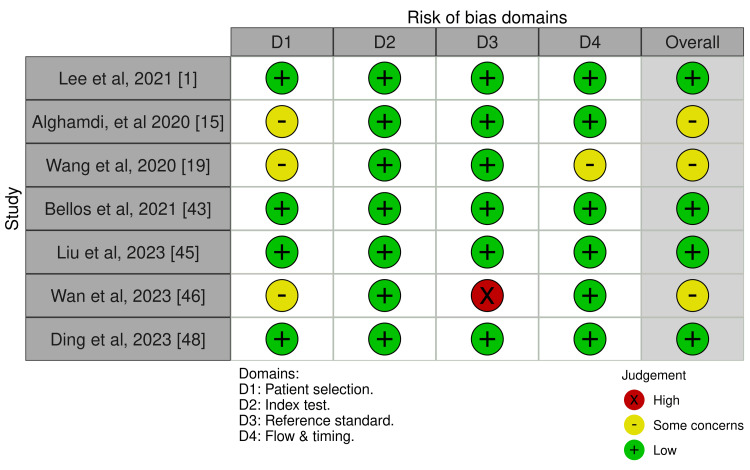
ROBIS tool for risk of bias assessment in systematic review and meta-analyses ROBIS: Risk of Bias in Systematic Reviews References: [1,15,19,43,45,46,48]

**Figure 4:**
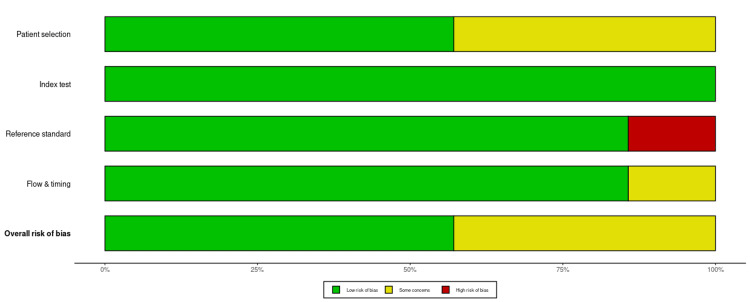
ROBIS tool for risk of bias assessment in sytematic reviews and meta-analyses included in the study ROBIS: Risk of Bias in Systematic Reviews

**Figure 5:**
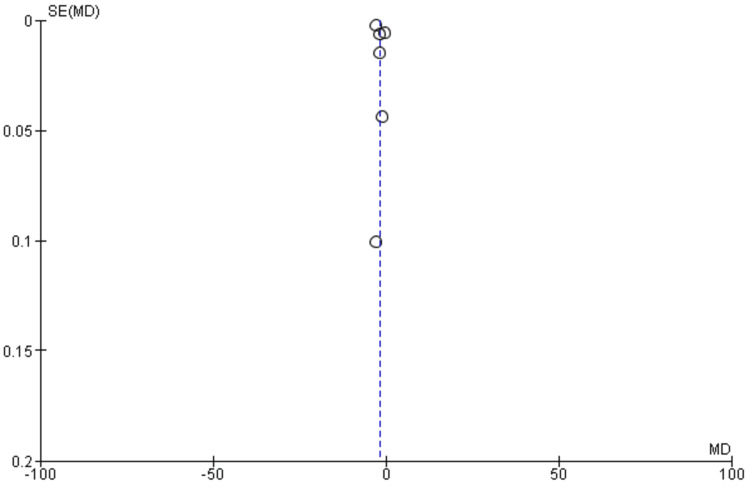
Funnel plot for meta-analyses included in the study

**Figure 6:**
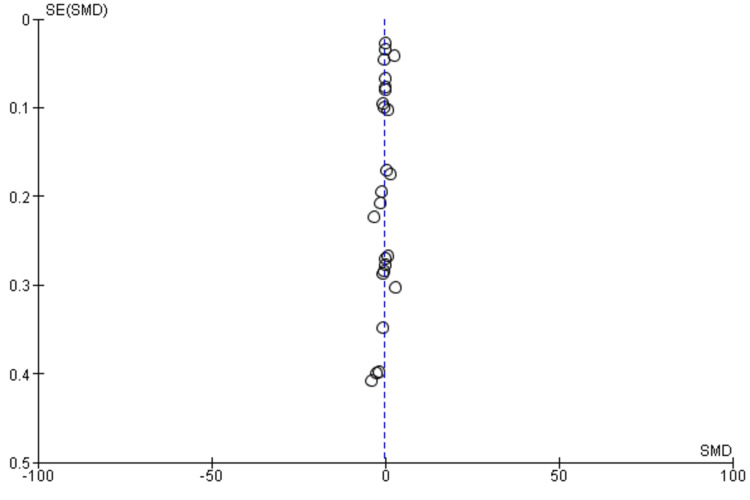
Funnel plot for the randomized controlled trials included in the study

The quality of evidence for the primary outcome was determined using the Risk of Bias in Systematic Reviews (ROBIS) assessment tool for systematic reviews and the RoB 2 assessment tool in RCTs (Figures 1-4) [17]. This framework includes five downgrade factors: limitations, inconsistency, indirectness, imprecision, and publication bias [18]. Most studies included in this meta-analysis were at a low risk of selection and reporting bias except a few studies where some concerns about the randomization and selection process were present. The funnel plots for both meta-analyses and RCTs included in this study showed a very low risk of publication bias as shown by funnel plots (Figure 5, 6).

Results

**Figure 7:**
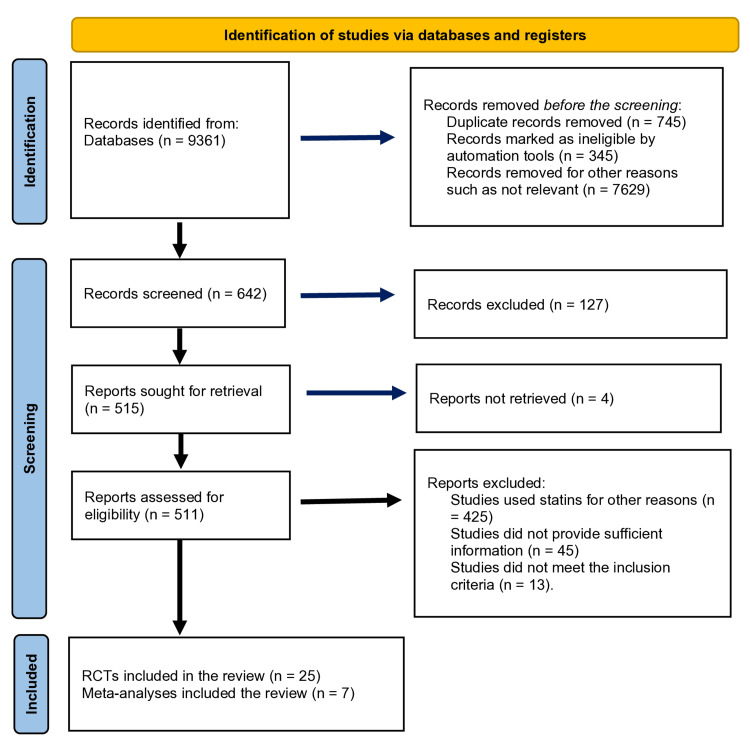
Preferred Reporting Items for Systematic Reviews and Meta-Analyses (PRISMA) 2020 flow diagram showing searches of databases and registers for current meta-analysis

Our literature search yielded 9361 studies in total and 8719 were excluded after screening the titles of these studies as the aims and objectives of these studies were different. Of the 642 studies, 127 examined other variables and did not examine the efficacy of statins for hypertension; hence, they were excluded from the meta-analysis. A total of 515 studies with preexisting hypertension were evaluated for inclusion in our meta-analysis by reading the full studies. Only 32 studies consisting of 25 RCTs and seven meta-analyses met the inclusion and exclusion criteria and were included in the final analysis. The literature search and selection of studies are shown in the Preferred Reporting Items for Systematic Reviews and Meta-Analyses (PRISMA) flow diagram (Figure 7).

The details of the studies included in this meta-analysis are presented in Tables 2, 3. Numerous articles have been published on the role of statins in hypertension and dyslipidaemia [1-48]. Table 2 provides demographic and outcome data for the 25 RCTs included in the current meta-analysis and Table 3 provides the data for the seven meta-analyses included in the current meta-analysis. The minimum follow-up duration for participants in these trials was two weeks [14] and the maximum follow-up duration was 60 months [40]. The number of participants in the RCTs' statin and placebo groups was 9678 and 9652, respectively. The minimum number of participants in a single RCT statin group was 13 [27] and the maximum number was 4126 [40]. The minimum number of participants in a single RCT placebo group was 13 [27] and the maximum number was 4159 [40].

**Table 2:** Demographic details and study findings for the randomized controlled trials included in this meta-analysis RCT: randomized controlled trial; OR: odds ratio; CI: confidence interval; PWV: pulse wave velocity; LDL-C: low density lipoprotein cholesterol; BMI: body mass index; ABP: ambulatory blood pressure; SBP: systolic blood pressure; DBP: diastolic blood pressure; ACEI: angiotensin-converting enzyme inhibitor; LVMI: left ventricular mass index

Study	Type of study	Study period	Number of patients in the statin group	Number of patients in control group	Reported outcome
Abebe et al., 2023 [13]	RCT	6 months	202	202	A significant reduction in mean systolic blood pressure from baseline (30.6 mmHg ± 18.7 vs. 25.24 mmHg ± 13.9, P = 0.001) and mean DBP reduction (20.4 mmHg ± 11.3 vs. 17.2 ± 9.0, P = 0.002) were observed in the statin group compared to the non-statin group at the end of 3 months. This difference was even higher at the end of 6 months in the statin group compared to the control group (32.9 mmHg ± 18.7 vs. 27.7 ± 14.0, P = 0.001 and 21.2 mmHg ± 11.0 vs. 18.1 ± 9.1, P = 0.002).
Ali et al., 2023 [14]	RCT	2 weeks	60	60	The study enrolled 120 hypertensive patients, and the mean systolic blood pressure and diastolic blood pressure were significantly lower in the combination therapy (Amlodipine and atorvastatin) group compared to amlodipine group only after two weeks of therapy(p≤0.05).
Beck et al., 2012 [20]	RCT	20 weeks	26	28	There was no difference in the baseline measurements and no change in BMI during the experimental period in either group. The statin group had significantly reduced LDL cholesterol and triglyceride levels at the end of weeks 8 and 20.
Cohn et al., 2009 [21]	RCT	2 months	337	331	Patients who received amlodipine and atorvastatin showed a greater reduction in both systolic and diastolic blood pressure at 8 weeks during this double control trial compared to the placebo group (P < 0.0001).
Correa et al., 2013 [22]	RCT	2 months	40	39	Simvastatin lowers ABP levels in patients with hypertension, particularly in the presence of high levels of cholesterol.
Danaoğlu et al., 2003 [23]	RCT	12 weeks	21	18	Patients in the treatment group received angiotensin-converting enzyme inhibitors (ACEI) and statins compared with statins only in the control group. Both groups showed a significant reduction in blood pressure.Systolic blood pressure (SBP) was reduced by 23% (p=0.0001), and diastolic BP was reduced by 23% (p=0.0001) compared to 20% (p=0.001) and 21% (p=0.001), respectively, in the control group. The pulse pressure (PP) decreased by 25% in the treatment group (P=0.0001) and 16% in the control group (p=0.0051).
Fogari et al., 2006 [24]	RCT	12 weeks	25	25	Hypertensive, obese, and normocholesterolemic patients in this trial showed a significant reduction in inflammatory markers, insulin resistance, and a decrease in systolic and diastolic blood pressure. The combination therapy with amlodipine-atorvastatin reduced systolic blood pressure by 22.5 mmHg and diastolic blood pressure by 17.7 mmHg as compared to placebo and amlodipine therapy alone.
Ge et al., 2008 [25]	RCT	4 months	61	65	Both groups experienced a reduction in both systolic and diastolic blood pressure (P < 0.05); however, the blood pressure in the combination therapy group was markedly lower than that in the amlodipine therapy group after treatment (P < 0.05). The left ventricular mass index (LVMI) decreased in both groups (P < 0.05); however, the reduction was greater in the combination therapy group (P < 0.05).
Golomb et al., 2008 [26]	RCT	6 months	618	309	Both simvastatin and pravastatin significantly reduced systolic and diastolic blood pressure relative to placebo. Blood pressure reductions ranged from 2.4 to 2.8 mm Hg for both systolic and diastolic blood pressures in both statin groups.
Gomes et al., 2010 [27]	RCT	3 weeks	13	13	Higher doses of atorvastatin were associated with a reduction in systolic and diastolic blood pressure reduction and muscle sympathetic nervous system activity compared to placebo.
Grimm et al., 2010 [28]	RCT	6 weeks	107	111	A total of 67.8% of patients in the amlodipine, atorvastatin, and therapeutic lifestyle (single combined pill) groups achieved a reduction in BP and LDL-C levels compared to only 9.6% in the amlodipine and lifestyle intervention groups at the end of 6 weeks. This effect was noticeable even at week 4 when 62.9% of participants attained the combined goal compared to amlodipine and therapeutic lifestyle changes by 5.2%.
Jin et al., 2020 [29]	RCT	8 weeks	131	66	The combined administration of telmisartan/amlodipine 80/5 mg and rosuvastatin 20 mg to treat hypertensive patients with dyslipidaemia significantly reduced blood pressure and improved lipid control.
Joyeux-Faure et al., 2014 [30]	RCT	3 months	25	26	A total of 51 patients were randomized to atorvastatin and placebo treatments in this study. The mean peripheral arterial tone (PAT) difference between the atorvastatin and placebo groups was 0.008 (−0.29; 0.28), P = 0.979. Both lipid profile and systolic blood pressure showed significant improvement in the atorvastatin group (mean difference: −6.34 mmHg (−12.68; −0.01), P = 0.050). Patients in the atorvastatin group also experienced improved endothelial function; however, carotid atherosclerosis and pulse wave velocity (PWV) remained unchanged in both groups.
Kamberi et al., 2012 [31]	RCT	12 months	38	24	Statins in combination with antihypertensive medications showed greater blood pressure reduction than antihypertensive medication alone.
Kanaki et al., 2011 [32]	RCT	26 weeks	25	25	Statins significantly reduced diastolic and systolic blood pressures in the treatment group compared to those in the placebo group. Statins showed a reduction in blood pressure during both the daytime and nighttime.
Koh et al., 2001 [33]	RCT	6 months	47	47	Simvastatin combined with losartan improved endothelial function and reduced inflammatory markers to a greater extent than monotherapy with either drug alone in hypercholesterolaemic or hypertensive patients.
Kushiro et al., 2009 [34]	RCT	60 months	1613	1664	Pravastatin was not associated with any significant blood pressure reduction, however, pravastatin resulted in significantly lower heart attacks and cerebrovascular accidents and it also reduced the burden of cardiovascular disease in patients with mild hypertension and hypercholesterolaemia.
Lavallée et al., 2009 [35]	RCT	3 months	45	46	There was no significant change in blood pressure control between statin and placebo groups and the mean absolute systolic blood pressure and diastolic blood pressure changes in the atorvastatin group were −3.9 mm Hg (95% CI, −8.2–0.3) and −2.8 mm Hg (95% CI, −5.5–−0.1), respectively. The corresponding changes for the placebo group were −0.8 mm Hg (95% CI, −5.2–3.6) and −1.4 mm Hg (95% CI, −4.2–1.5) respectively.
Lewandowski et al., 2010 [36]	RCT	8 weeks	15	16	There were no obvious changes in either systolic or diastolic blood pressure or plasma levels of catecholamines, neuropeptide Y, endothelin, aldosterone, and renin activity. In patients with hypertension and hypercholesterolaemia, simvastatin reduces muscle sympathetic nerve activity.
Mancia et al., 2010 [37]	RCT	30 months	253	254	The co-administration of statins and antihypertensive medication in patients already receiving antihypertensive treatment did not result in significant blood pressure reduction.
Manisty et al., 2009 [38]	RCT	12-18 months	64	64	There was a mild reduction in local wave velocity which is a measure of carotid artery stiffness, in the atorvastatin group; however, this was not significant. Patients receiving combination therapy of antihypertensives and statins showed a reduction in blood pressure compared to the placebo group.
Sever et al., 2009 [39]	RCT	3.3 years	2584	2554	Atorvastatin reduced coronary heart disease deaths and nonfatal myocardial infarction by 46% [hazard ratio 0.54, confidence interval (CI) 0.40-0.72], stroke by 37% [hazard ratio 0.63, CI 0.46-0.87] and total cardiovascular events by 27% in the blood pressure lowering arm assigned to receive amlodipine with atorvastatin and in the group assigned to receive In the atenolol with atorvastatin, it reduced coronary heart disease death and nonfatal myocardial infarction by 25% [hazard ratio 0.75, CI 0.57-0.97], stroke by 10% [hazard ratio 0.90, CI 0.69-1.18] and total cardiovascular events by 13%.
Tonelli et al, 2006 [40]	RCT	60 months	4126	4159	There was no statistically significant difference in blood pressure control between the pravastatin and placebo groups during the follow-up period. Pravastatin treatment did not reduce the adjusted risk of systolic or diastolic hypertension (OR 0.99, 95% CI 0.80–1.23) for systolic hypertension and (odds ratio 0.97, 95% CI 0.73–1.27) for diastolic hypertension.
William et al., 2009 [41]	RCT	3.5 years	434	457	Statins were found to have no positive effect on reducing blood pressure in this trial compared with placebo. The change in brachial blood pressure in the statin and placebo groups was -0.1 mm Hg [95% CI, -1.8 to 1.6], P=0.9 and change in the brachial pulse pressure was -0.02 mm Hg [95% CI, -1.6 to 1.6], P=0.9.
Zaleski et al., 2014 [42]	RCT	6 months	202	217	In this trial, women taking atorvastatin showed a reduction in systolic and diastolic blood pressure from baseline over 6 months, whereas this effect was not observed in men taking atorvastatin. These sex-dependent blood pressure effects of atorvastatin were not different from those of the placebo.

**Table 3:** Demographic findings for the meta-analyses included in this study SBP: systolic blood pressure; DBP: diastolic blood pressure; MD: mean difference; CI: confidence interval; OR: odds ratio; RR: relative risk; BP: blood pressure

Study	Type of study	Study period	Number of patients in the statin group	Number of patients in control group	Reported outcome
Lee et al., 2021 [1]	Meta-analysis	NA	94,466	15,228	SBP decreased in the rosuvastatin or pravastatin subgroup, and DBP decreased in the simvastatin or pravastatin subgroup
Alghamdi et al., 2020 [15]	Meta-analysis	NA	24,589	24,498	The meta-analysis showed that statin reduced systolic BP by - 1.6 mmHg (95% CI: - 2.50 to - 0.60), and diastolic BP by - 0.96 mmHg (95% CI: - 1.36 to - 0.56). This effect was independent of the dose or type of statin used (p > 0.05).
Wang et al., 2020 [19]	Meta-analysis	NA	288	219	Rosuvastatin could be beneficial in controlling hypertension.
Bellos et al., 2021 [43]	Meta-analysis	2 to 72 months	22 867	22 856	The mean difference for systolic blood pressure was –1.42 (95% CI: –2.38, –0.46; p = .004) and diastolic blood pressure was 0.82 (95% CI: − 1.28, − 0.36; p = .0005). The observed decreases in both SBP and DBP in both groups were not large enough to be considered significant.
Liu et al., 2023 [45]	Meta-analysis	36 to 72 months	19656	18962	The meta-analysis included eight randomised controlled trials with 38,618 patients, and the results showed significantly reduced major adverse cardiovascular events in patients on combination therapy compared to antihypertensive therapy alone (RR, 0.79; 95% CI 0.71–0.88; p < 0.001). Furthermore, there were fewer events of myocardial infarction (RR, 0.67; 95% CI, 0.53–0.84; p = 0.001) and stroke risks (RR 0.82; 95% CI 0.72–0.94; p = 0.005) in patients on combination therapy; however, there was no difference in all-cause mortality between the two groups (RR, 0.95; 95% CI, 0.86–1.04; p = 0.277).
Wan et al., 2023 [46]	Meta-analysis	NA	1553	1533	The study results showed that the statin group was superior to the placebo group in terms of systolic blood pressure, with a mean difference (MD) of 4.37, 95% CI [0.72, 8.02], p = .02, p = .02, I2 = 99%. However, there was no difference in diastolic blood pressure between the statin and placebo groups (MD = 2.48, 95% CI [−2.00, 6.96], p = .28, random effects model). The funnel plot showed very little evidence of publication bias.
Ding et al., 2023 [48]	Meta-analysis	NA	2250	2200	The combination therapy of amlodipine and statins resulted in a significantly greater percentage reduction in systolic blood pressure in patients (MD= −2.22%, 95% confidence intervals: [−3.82 to −0.62]). Combination therapy also resulted in a significant reduction in low-density lipoprotein cholesterol levels.

Most studies demonstrated a positive effect of statins in hypertensive patients, either alone or as a combination therapy, and this effect was evident both during the day and night. Statins were effective in reducing both SBP and DBP in patients, although the exact effects varied between studies. It is important to mention that a lot of variation was observed in the methodology of the included studies. Additional benefits of statin therapy observed in these studies include improved endothelial function and reduction in inflammatory response and LDL cholesterol (LDL-c) levels. Studies using a combination therapy of statins with antihypertensive medications showed better BP control than antihypertensive therapy alone. The number of patients included in the RCTs measuring DBP in the statin and control groups was 8713 and 8874, respectively.

The total number of patients in the meta-analyses' statin and control groups was 145,958 and 66,485, respectively. Individual study demographics and results are presented in Tables 2 and Table 3, respectively. Studies have shown that statins effectively reduce both SBP and DBP in patients when used alone or combined with antihypertensive therapy. Abebe et al. reported a mean difference of 5 mmHg in SBP and 3 mmHg in DBP in the statin group after six months of therapy [13]. Similarly, Danaoğlu et al. reported a 23% decline in SBP in the statin and ACEI groups compared to 20% in patients on ACEI and placebo only [23].

**Figure 8:**
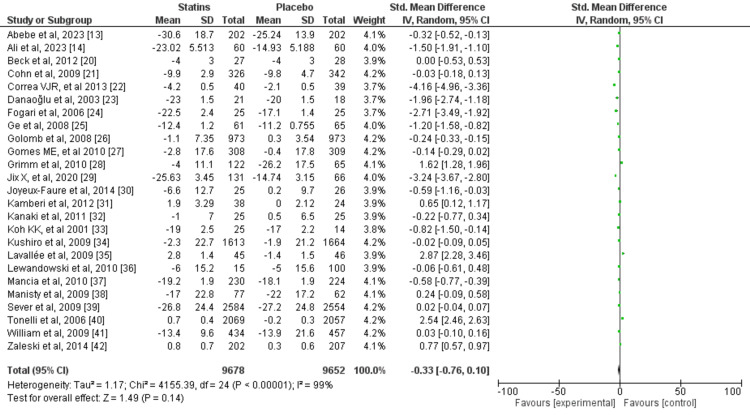
Systolic blood pressure forest plot for randomized controlled trials included in the meta-analysis References: [13,14,20-42]

**Figure 9:**
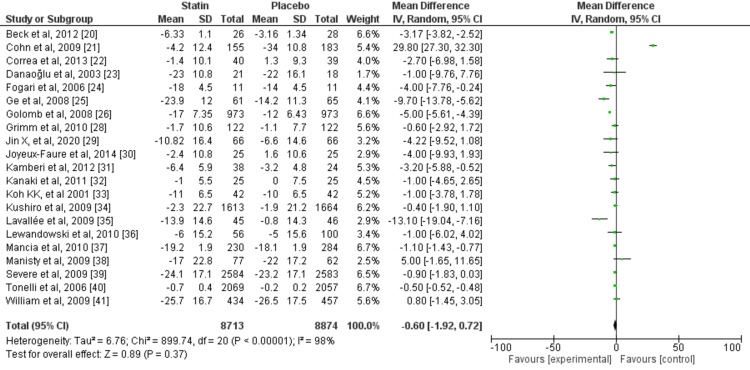
Diastolic blood pressure forest plot for the randomized controlled trials included in the meta-analysis References: [20-41]

**Figure 10:**
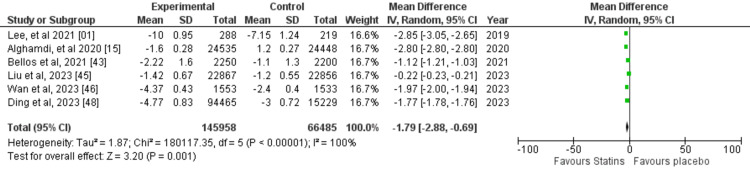
Forest plot for meta-analyses included in this study. References: [1,15,43,45,46,48]

A random-effects model was used for the meta-analysis due to heterogeneity in the studies. The mean difference (MD) for RCTs measuring SBP was -0.33. There was a significant variation in the true effect size across the studies, as shown by a Tau2 value of 1.17, and significant heterogeneity was observed across the studies, with an I2 value of 99% (Figure 8). Similarly, the MD for RCTs measuring diastolic blood pressure was -0.60, there was a significant between-study variance Tau2 value of 6.76 and I2 for heterogeneity was 98%, demonstrating considerable heterogeneity (Figure 9). The MD for the included meta-analyses was -1.79 and the true effect size variation between studies was Tau2 1.87. Considerable heterogeneity was observed across the studies, with an I2 value of 100% (Figure 10). A meta-analysis based on RCTs demonstrated a synergistic effect of statins on both SBP and DBP.

Discussion

Several studies, including RCTs and meta-analyses, have demonstrated the blood pressure-lowering effects of statins when used alone or in combination with antihypertensive therapy in patients with hypertension [1-48]. Statins also offer additional benefits, such as cholesterol-lowering effects, improved endothelial function, and reduced inflammatory response. Statins reduce SBP and DBP, as shown in previous trials, although a few trials did not support these findings. The Atorvastatin and Amlodipine in Patients With Elevated Lipids and Hypertension (AVALON) Arterial Wall Compliance trial, which used a combination therapy of amlodipine and statins, demonstrated a reduction in cardiovascular events and a 19% improvement in small artery compliance compared with amlodipine therapy alone [21]. This trial was a substudy of the larger AVALON trial, which investigated the safety and efficacy of co-administered statins and amlodipine therapy in patients with hypertension and dyslipidaemia.

A meta-analysis showed that statins were more effective in reducing BP in patients with higher baseline BP [49]. The effects of statins on SBP and DBP in this study when restricted to studies with baseline SBP > 130 mmHg and DBP > 80 mmHg were -4.0 mmHg and -1.2 mmHg, respectively. This effect was negligible when only trials with baseline SBP < 130 mmHg and DBP < 80 mmHg were included in the meta-analysis. Meta-regression analysis did not show any effects of variables such as age, diabetes, use of antihypertensive therapy, cholesterol level, and duration of the trial [49].

There are several possible mechanisms through which statins may affect BP. Experimental studies have shown that statins increase endothelial production of nitric oxide which is correlated with the upregulation of endothelial nitric oxide synthase expression, resulting in the simultaneous inhibition of G proteins. This leads to reduced endothelial nitric oxide synthase messenger ribonucleic acid (mRNA) degradation and increased nitric oxide bioavailability. Another possible mechanism by which statins affect BP is reduced arterial stiffness and improved systemic arterial compliance. This in turn leads to alterations in the relative content of arterial vascular smooth muscle cells and the restoration of endothelial function restoration [49,50]. A further possible explanation for this mechanism could be the downregulation of angiotensin II-type 1 receptor by statins, which is overexpressed in hypercholesterolaemic patients. This alteration is corrected by statins which markedly reduce the vasoconstrictor response to angiotensin II infusion [49,51]. Statins are known to have cardioprotective effects by reducing the incidence of CVD through their cholesterol-lowering mechanism, anti-inflammatory role, and BP-lowering effects [46].

The studies included in the current meta-analysis were mainly RCTs, and the included meta-analyses were also based on RCTs only. Our study also confirmed the findings of previous studies that statins alone or in combination have a BP-lowering effect. The risk of publication bias was also very low in these studies, as shown by the funnel plots. A previous meta-analysis that included 40 studies and 45,113 patients showed a small but significant reduction in SBP and DBP [52]. The antihypertensive effect of statins was noted to be independent of age, trial length, or changes in serum cholesterol levels [49]. A major drawback of most of these trials is that they failed to investigate the clinical endpoints after long-term statin therapy [19]. Another meta-analysis based on 12 RCTs showed that patients receiving statins had reduced cardiovascular morbidity and mortality, and this association was independent of the patient's BP status [47]. A meta-analysis of 65,000 patients on the use of statins in primary prevention found that statins were effective in reducing mortality and morbidity irrespective of age and sex [53]. To date, there is no evidence that one class or group of statins is more effective than the other, and the only trials that performed head-to-head trials on statins were the Pravastatin or Atorvastatin Evaluation and Infection Therapy (PROVE-IT) and Atorvastatin versus Simvastatin on Atherosclerosis Progression (ASAP) trials that did not show any difference between individual statins [54,55]. One meta-analysis did not find any difference between individual statins among placebo trials, but differences were noted between individual statins when combined with usual care controls [56].

A meta-analysis by Sundström et al. suggested that statins and antihypertensive therapy provided multiple benefits in terms of cardiovascular outcomes; however, this study did not directly compare the cardiovascular outcomes between combination therapy and antihypertensive therapy alone [57]. Our meta-analysis also showed similar cardioprotective and BP-lowering effects of statins alone and in combination therapy.

## Conclusions

This meta-analysis supports the findings of previous studies demonstrating the BP-lowering effect of statins, along with other cardiovascular benefits. Statins also improve endothelial function and have anti-inflammatory roles in addition to lipid-lowering effects. It is unclear whether statins lower BP in patients with hypercholesterolaemia and normotension, and further research in this direction would be useful. Statins play a clear role in both the primary and secondary prevention of cardiovascular events, depending on the individual risk. A large-scale multicentre RCT focusing on the type and intensity of statins for the prevention of major cardiovascular events is recommended.
